# MiR-125a suppresses tumor growth, invasion and metastasis in cervical cancer by targeting STAT3

**DOI:** 10.18632/oncotarget.4457

**Published:** 2015-07-13

**Authors:** Zhongyi Fan, Hanzhi Cui, Xiaojie Xu, Zhi Lin, Xuelin Zhang, Lei Kang, Baiyu Han, Jing Meng, Zhifeng Yan, Xiang Yan, Shunchang Jiao

**Affiliations:** ^1^ Department of Oncology, PLA General Hospital, Beijing, China; ^2^ Department of Oncology, 309^th^ Hospital of PLA, Beijing, China; ^3^ Department of Medical Molecular Biology, Beijing Institute of Biotechnology, Beijing, China; ^4^ Department of Nuclear Medicine, Peking University First Hospital, Beijing, China; ^5^ Department of Endocrinology and Metablism, 264^th^ Hospital of PLA, Shanxi, China

**Keywords:** miR-125a, cervical cancer, cell growth, metastasis, STAT3

## Abstract

MiR-125a has been characterized as a tumor suppressor in several cancers. However, the role of miR-125a in cervical cancer is unknown. In this study, we found the expression of miR-125a was downregulated in cervical cancer patients, and negatively correlated with the tumor size, FIGO stage, and preoperative metastasis. Kaplan-Meier analysis showed that miR-125a expression predicted favorable outcome for cervical cancer patients. Dual luciferase assays identified the STAT3 gene as a novel direct target of miR-125a. Functional studies showed that miR-125a overexpression significantly suppressed the growth, invasion and epithelial-mesenchymal transition (EMT) of cervical cancer cells both *in vitro* and *in vivo* via decreasing STAT3 expression. Moreover, miR-125a conferred to G2/M cell cycle arrest, accompanied by inhibition of several G2/M checkpoint proteins. Mechanistically, inactivation of miR-125a during cervical carcinogenesis was caused by HPV suppression of p53 expression. Clinically, STAT3, the expression of which, predicted poorer outcome, was inversely correlated with miR-125a in cervical cancer. These data highlight the importance of miR-125a in the cell proliferation and progression of cervical cancer, and indicate that miR-125a may be a useful therapeutic target for cervical cancer.

## INTRODUCTION

Cervical cancer (CC) is a commonly diagnosed gynecological cancer, and the third most common cancer and fourth leading cause of cancer-related death among women worldwide [1, 2]. To date, there are 529 800 new cases and 275 100 deaths per year, accounting for approximately 9% of all female cancer incidence and mortality [3]. More than 80% of these cases are estimated to occur in developing countries [4]. Radiotherapy, chemotherapy, and surgery are standard treatments for CC, but the 5-year survival rate for advanced patients remains very low [5]. Metastasis to the lymph node and distant organs is a major cause of treatment failure [6]. Thus, elucidation of the molecular mechanisms underlying CC tumorigenesis and progression is critical for individualized treatments of CC. It is widely accepted that CC development is related to human papillomavirus (HPV) infection [7, 8]. However, HPV is necessary but not sufficient to cause cervical carcinoma. Thus, other factors must contribute to CC development [9, 10], including abnormal expression of multiple genes [11–13]. However, our understanding of the genetic alterations underlying the development of CC remains limited.

MicroRNA (miRNA)-mediated regulation of post-transcriptional gene expression has been recently identified in the development and metastasis of many cancer types including CC [14, 15]. MiRNAs are endogenous non-coding short RNAs that inhibit gene expression by binding to target mRNAs at their 3′-untranslated region (UTR) [16]. They have been implicated as oncogene or anti-oncogene of several diseases including cancer [17, 18]. Differential expression of miRNAs has been found in cancer and adjacent tissues, suggesting their potential applications as biomarkers and therapeutic targets [18]. Previous studies have indicated that miR-125a is a novel anti-oncogene with low expression in several cancers [19–21]. However, the association between miR-125a and CC remains unclear.

In this study, we found lower expression of miR-125a in CC tissues than in matched non-cancerous tissues. Furthermore, miR-125a inhibits CC cell proliferation and invasion, while suppresses their cell cycle. MiR-125a overexpression reduces cervical tumor growth and metastasis *in vivo*. Moreover, miR-125a regulates these events by directly binding STAT3 3′-UTR and inhibiting its expression and downstream genes. Thus, overexpression of miR-125a may be a useful strategy for the treatment of CC patients with hyperactivation of STAT3.

## RESULTS

### Expression of miR-125a and its correlation with clinical parameters in CC patients

**Figure 1:**
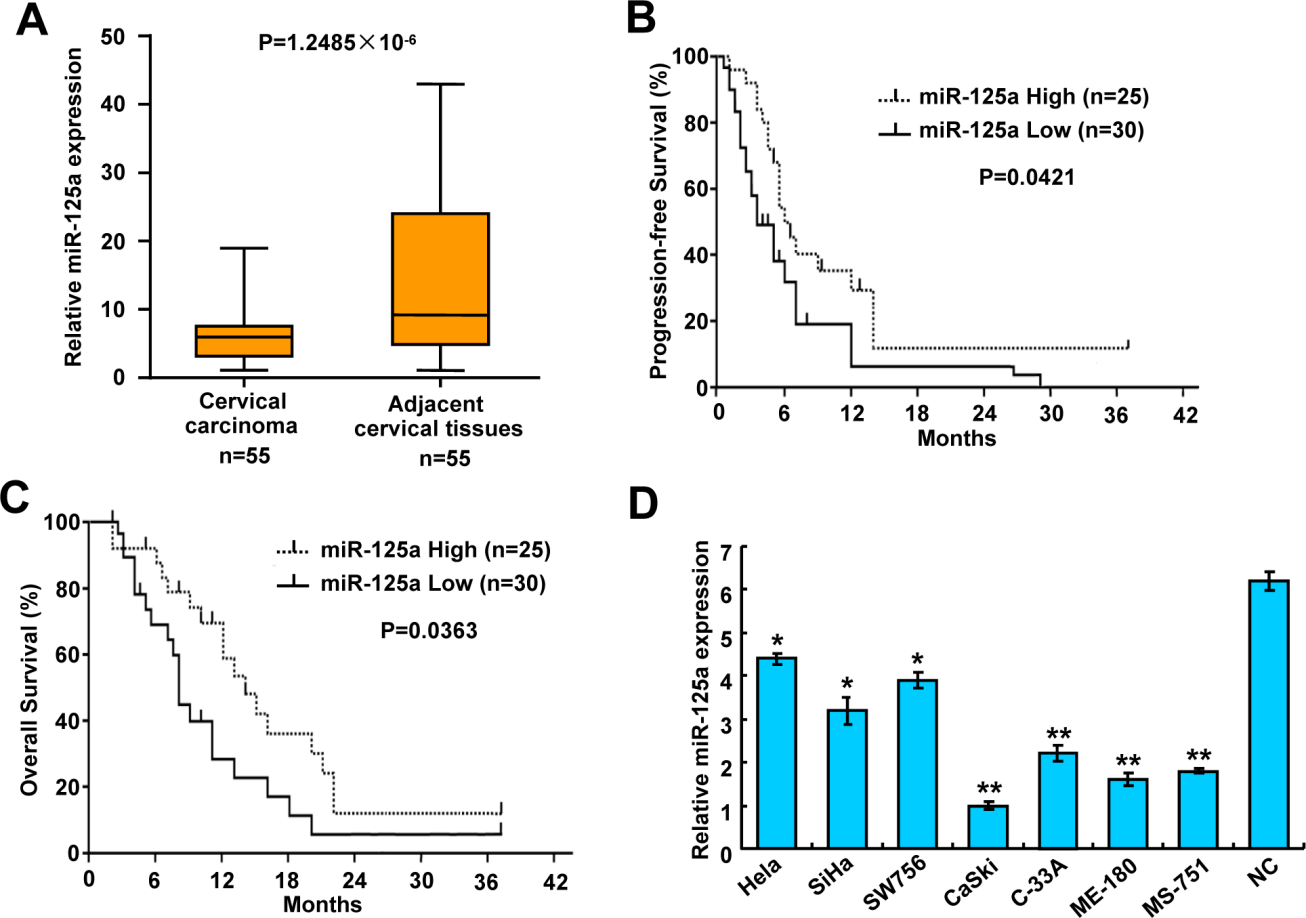
Expression of miR-125a in CC tissues and cell lines, and the correlation between miR-125a and clinical parameters in CC patients **A.** Expression of miR-125a in CC tissues and matched adjacent normal cervical tissues (*n* = 55) was compared using the Mann-Whitney *U*-test. *U6* small nuclear RNA was used as an internal control. **B** and **C.** Kaplan-Meier survival curves and the log-rank test were used to assess (C) PFS and (D) OS compared between low and high expression of miR-125a in CC patients. **D.** Expression of miR-125a in seven CC cell lines (Hela, SiHa, SW579, CaSki, C-33A, ME-180, and MS-751) and normal cervical epithelial cells. All values are the mean ± SD of triplicate measurements, and experiments were repeated 3 times with similar results. *P < 0.05, **P < 0.01 compared to normal cervical cells.

To investigate the clinical significance of miR-125a in CC, we first detected the expression of miR-125a by qRT-PCR in 55 pairs of cervical tumors and matched non-tumor cervical tissues. Compared with their corresponding non-tumorous counterparts, miR-125a expression was significantly downregulated in cervical tumor tissues (*P* = 1.2485 × 10^−6^) (Figure 1A and Supplementary Figure 1). To further investigate the relevance between miR-125a and clinicopathological characteristics, we divided the cervical tumor samples into two groups according to their miR-125a expression levels. As expected, the low miR-125a expression group showed higher incidences of larger tumors sizes (*P* = 0.016), late FIGO (International Federation of Gynecology and Obstetrics) stages (*P* = 1.328 × 10^−4^), and preoperative metastasis (*P* = 0.001). However, no significant differences were observed in terms of age, SCC-Ag, and tumor histology (Table 1). Moreover, Kaplan–Meier survival analysis revealed that patients with low miR-125a expression had poorer progression-free survival (PFS) (*P* = 0.0421) and overall survival (OS) (*P* = 0.0363) than those with high miR-125a expression (Figure 1B and 1C). To investigate the role of miR-125a in CC, we examined its expression in CC cell lines and normal cervical epithelial cells. Expression of miR-125a was lower in CC cells compared with that of normal cervical epithelial cells. In addition, miR-125a expression was lower in four cell lines derived from metastatic sites than in three cell lines derived from primary cervical cancers (Figure 1D). Taken together, these findings indicate that miR-125a correlates CC prognosis, tumor growth, and metastasis.

**Table 1:** Clinical correlations of miR-125a expression in cervical carcinoma

Variables	miR-125a expression in tumor tissues (T)			*P* value
	Low expression	High expression	Total	
	*N* = 30 (54.5%)	*N* = 25 (45.5%)	*N* = 55 (100%)	
Age (mean± SD), years	53.5 ± 9.59	55.48 ± 9.89	54.4 ± 9.69	0.866
Tumor size				
<4 cm	4 (26.7%)	11 (73.3%)	15	0.016^†^
≥4 cm	26 (65%)	14 (35%)	40	
SCC-Ag				
<1.5 ng/ml	23 (60.5%)	15 (39.5%)	38	0.245
≥1.5 ng/ml	7 (41.2%)	10 (58.8%)	17	
Histology				
Adenocarcinoma	2 (40.0%)	3 (60.0%)	5	0.650
Squamous	28 (56.0%)	22 (44.0%)	50	
FIGO Stage				
I/II	7 (26.9%)	19 (73.1%)	26	1.328 × 10^−4^^†^
III/IV	23 (79.3%)	6 (20.7%)	29	
Preoperative metastasis§				
Absent	8 (52.8%)	18 (47.2%)	26	0.001^†^
Present	22 (32.4%)	7 (67.6%)	29	

*P*-values of age were calculated by the *t*-test, others by Pearson's chi-square test.

† Statistically significant.

§ Preoperative metastasis indicates preoperative local lymphatic metastasis and distant metastasis.

### MiR-125a inhibits STAT3 expression by binding its 3′-UTR

**Figure 2:**
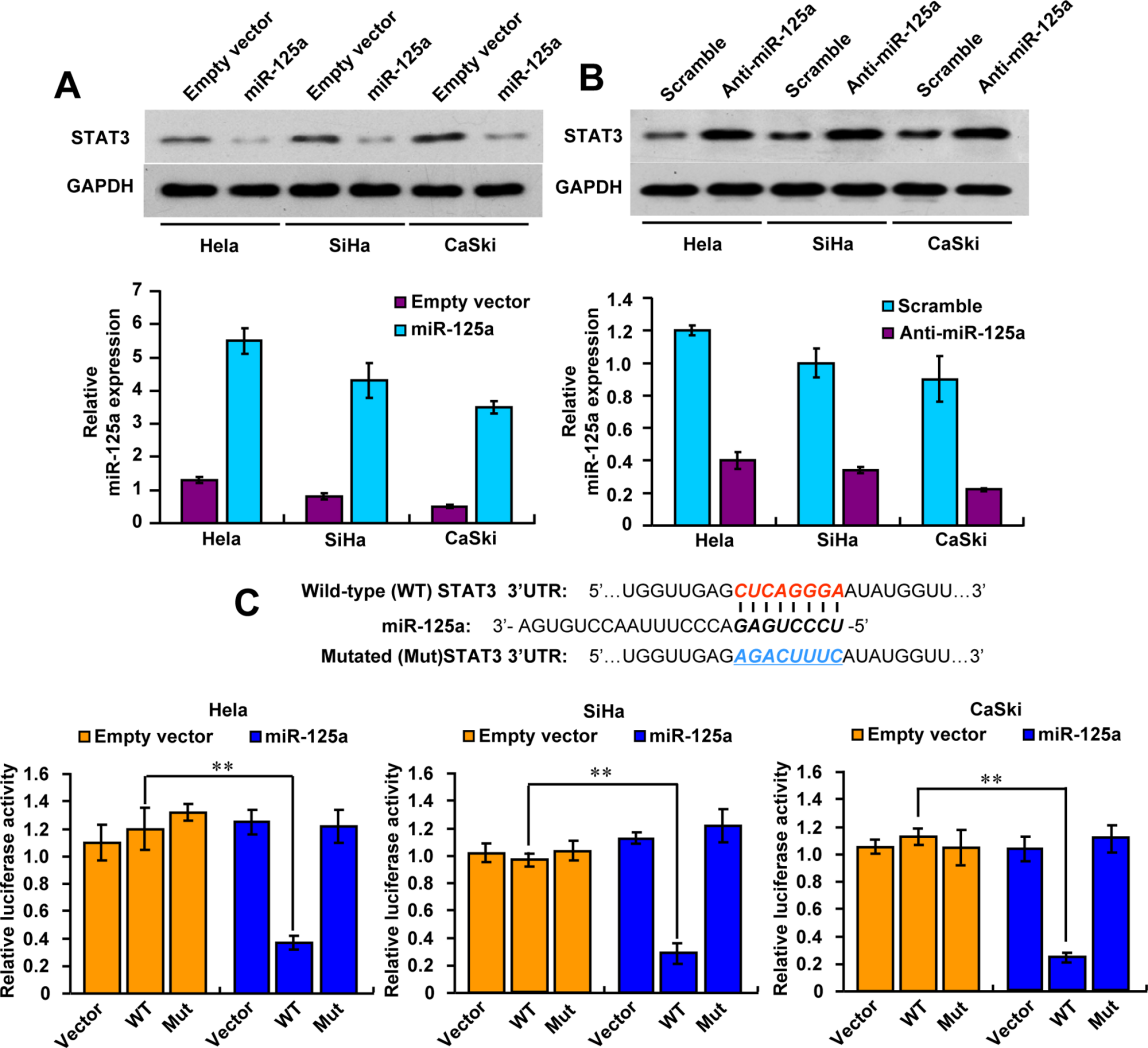
MiR-125a suppresses STAT3 expression by targeting its 3′-UTR **A** and **B.** Immunoblot analysis of the indicated CC cell lines transfected with (A) miR-125a or (B) anti-miR-125a. Histograms under the immunoblots show the corresponding miR-125a mRNA expression levels. **C.** MiRNA luciferase reporter assays in Hela, SiHa, and CaSki cells transfected with wild-type or mutated STAT3 reporters plus miR-125a. The top panel indicates wild-type and mutant forms of putative miR-125a target sequences in the STAT3 3′-UTR. Bold and italicized fonts indicate putative miR-125a-binding sites in the human STAT3 3′-UTR. Underlining indicates mutations introduced into the STAT3 3′-UTR. All values shown are the mean ± SD of triplicate measurements. Experiments were repeated three times with similar results. (**P < 0.01)

To investigate the mechanisms responsible for the functions of miR-125a in CC, we searched for candidate target genes of miR-125a using publicly available databases (TargetScan and miRanda). Considerable genes were predicted as the potential targets of miR-125a, and we picked out those reported to play a role in CC (Supplementary Figure 2A). Next, we performed Western blot analysis to identify those potential targets in Hela cells successively, and finally found that only the protein level of STAT3 could be decreased by miR-125a, which indicated that STAT3 was the target gene of miR-125a (Supplementary Figure 2B). In agreement with the results in Hela cells, we also found that miR-125a inhibited STAT3 expression in SiHa and CaSki cells (Figure 2A). In contrast, miR-125a inhibitor increased STAT3 expression in those cell lines (Figure 2B). qRT-PCR was performed to confirm the expression levels of miR-125a after overexpression or knockdowned (Figure 2A and 2B).

To confirm whether STAT3 is a direct and specific target of miR-125a, the STAT3 3′-UTR or mutant 3′-UTR luciferase reporters were co-transfected with the expression plasmid. Luciferase reporter assays showed that miR-125a decreased STAT3 3′-UTR reporter activity by more than 60% in Hela, SiHa and CaSki cells, but not mutant 3′-UTR reporter activity with mutations in the binding sites for miR-125a (Figure 2C). Taken together, these results indicate that miR-125a inhibits STAT3 expression by directly binding its 3′-UTR in CC cells.

### MiR-125a suppresses CC cell proliferation through inhibition of STAT3 expression

**Figure 3:**
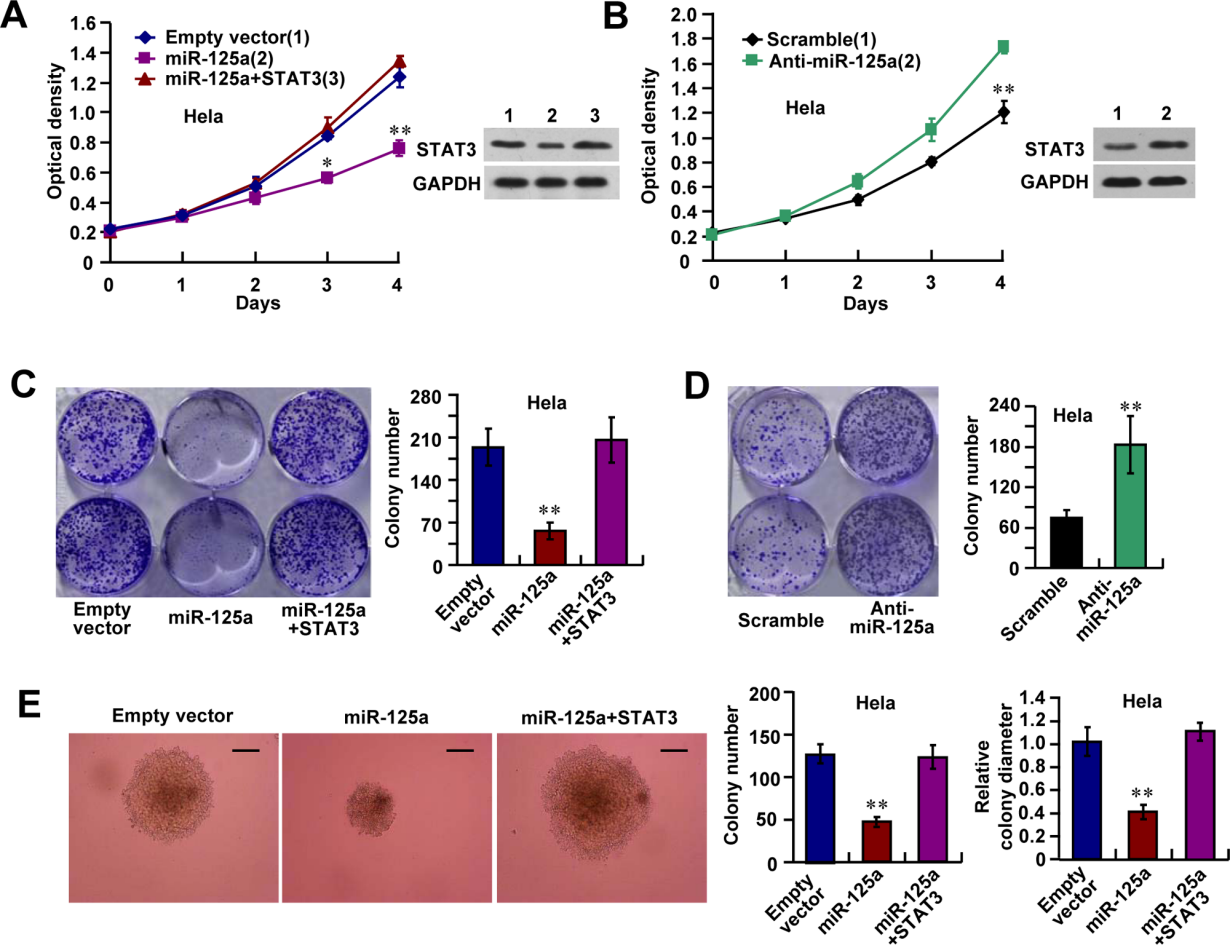
miR-125a suppresses cell proliferation through inhibition of STAT3 expression Hela cells were transfected with (A, C, and E) miR-125a, miR-125a plus STAT3, or (B and D) anti-miR-125a. **A** and **B.** Cell growth assays were performed using a CCK-8 kit at the indicated times. (A and B right top panels) Representative immunoblots showing STAT3 expression. **C** and **D.** Colony formation assays using Hela cells. Histograms show the colony number. **E.** Hela cells were plated in soft agar and assayed for the colony number and comparison of colony diameters. Representative images show colonies in soft agar (left panels). All values shown are the mean ± SD of triplicate measurements. Experiments were repeated three times with similar results. (*P < 0.05, **P < 0.01)

To investigate the biological functions of miR-125a in CC cells, Hela cells were transfected with miR-125a and then subjected to cell growth analyses. Cell proliferation and colony formation assays revealed that miR-125a overexpression reduced the proliferative ability of Hela cells (Figure 3A and 3C), whereas inhibition of miR-125a enhanced the proliferation of Hela cells (Figure 3B and 3D). Introduction of STAT3 reversed the effect of miR-125a on Hela cell proliferation (Figure 3A and 3C). Similar effects were observed in SiHa and CaSki cell lines (Supplementary Figure 3A–3D). We also found that suppression of miR-125a inhibited anchorage-independent proliferation of Hela cells in soft agar assays (Figure 3E). Again, overexpression of STAT3 reversed the effect of miR-125a on anchorage-independent proliferation of Hela cells (Figure 3E). These results suggest that miR-125a impairs the growth of CC cells via downregulation of STAT3.

### MiR-125a induces cell cycle arrest and down-regulation of c-myc in CC cells

**Figure 4:**
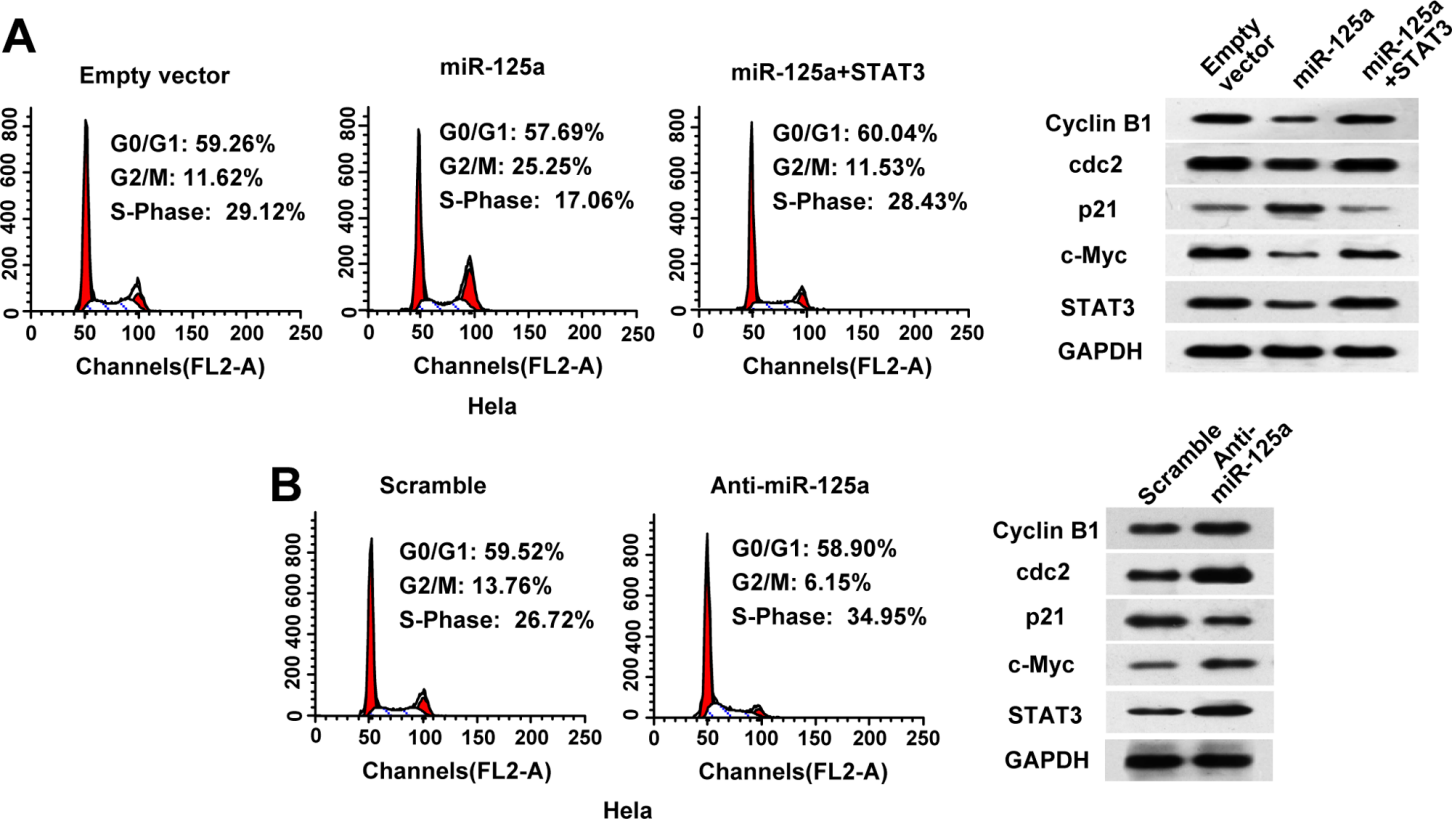
MiR-125a induces cell cycle arrest at G2/M transition in CC cells through suppression of STAT3 **A.** Flow cytometric analysis of the cell cycle in Hela cells transfected with the empty vector, miR-125a, or miR-125a plus STAT3. **B.** Flow cytometric analysis of the cell cycle in Hela cells transfected with scramble or anti-miR-125a. Experiments were repeated three times with similar trends. Representative data from one experiment are shown. (A and B right panels) Representative western blots of cyclin B1, cdc2, p21, c-myc, and STAT3 proteins in the indicated Hela cells.

To investigate the mechanisms of miR-125a suppression in CC cell proliferation, we examined the effects of miR-125a on the cell cycle by flow cytometry. Compared with control cells, overexpression of miR-125a in Hela cells significantly increased the proportion of cells in G2/M phase (from 11.62% to 25.25%) (Figure 4A). Furthermore, overexpression of miR-125a inhibited the expression of G2/M phase checkpoint proteins cyclin B1 and cdc2, and increased the expression of p21 (Figure 4A). In contrast, inhibition of miR-125a in Hela cells led to a reduction in the proportion of cells in G2/M phase (from 13.76% to 6.15%) (Figure 4B), along with increased expression of cyclin B1 and cdc2, and decreased expression of p21 (Figure 4B). Re-expression of STAT3 reversed the effects of miR-125a on the cell cycle and G2/M checkpoint proteins (Figure 4A). Similar effects were observed in SiHa cells (Supplementary Figure 4A and 4B). These data suggest that miR-125a leads to cell cycle arrest at G2/M transition in CC cells.

Moreover, we found that miR-125a inhibited expression of the oncogene c-myc through STAT3 in Hela and SiHa cells (Figure 4A and 4B, and Supplementary 4A and 4B). These data suggest that miR-125a inhibits cell proliferation also through the suppression of c-myc expression.

### MiR-125a suppresses CC cell invasion through inhibition of STAT3-related matrix metalloproteinase (MMP)-2, MMP-9 and EMT markers expression

**Figure 5:**
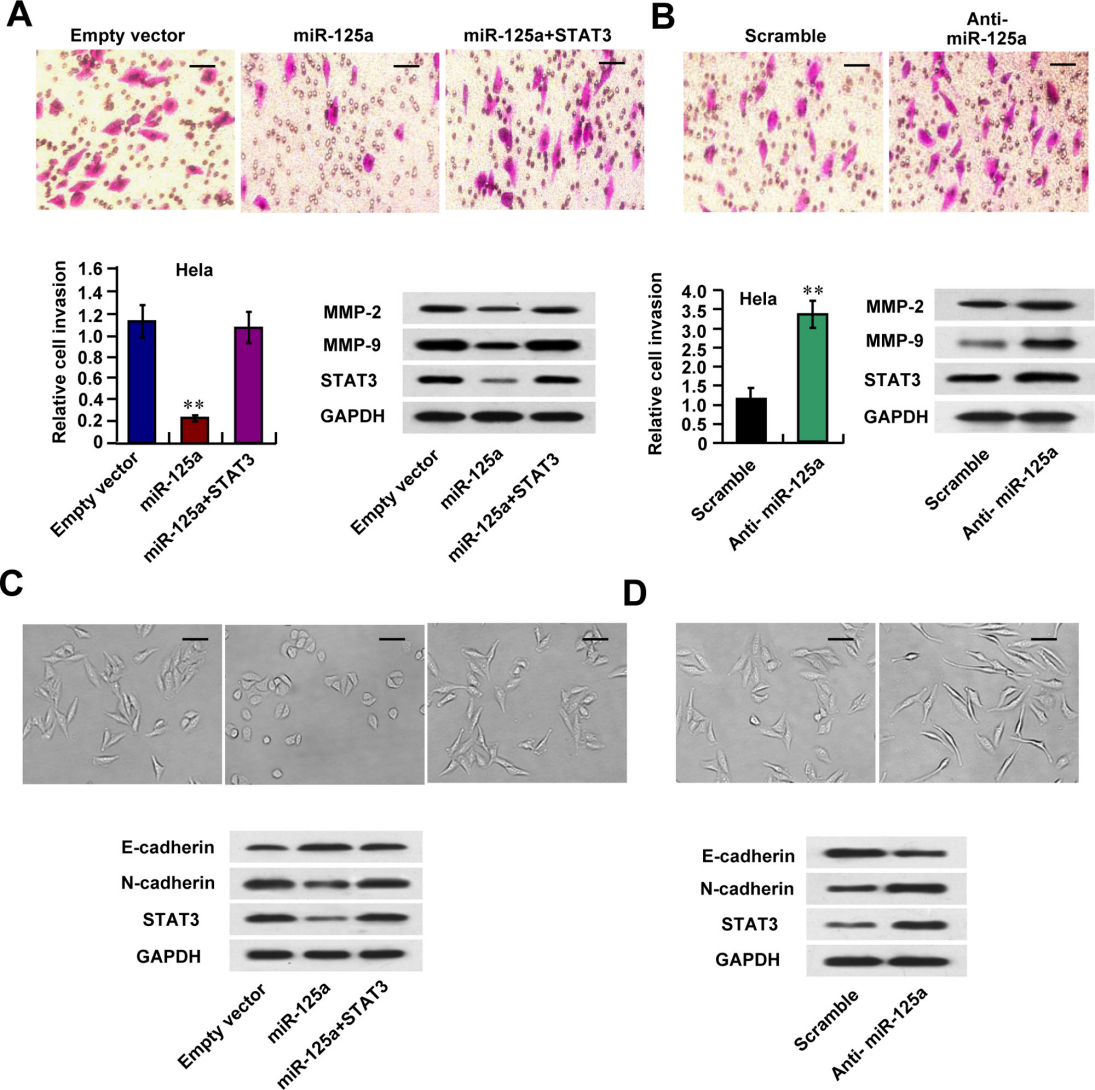
miR-125a inhibits invasion of CC cells via down-regulation of MMP-2, MMP-9 expression and EMT Hela cells were transfected with (A and C) miR-125a, miR-125a plus STAT3, or (B and D) anti-miR-125a. **A** and **B.** Cell invasion of Hela cells was evaluated using a Matrigel invasion chamber. Invaded cells were fixed and stained with crystal violet (A and B top images). Western blots of MMP-2, MMP-9, and STAT3 proteins in the indicated Hela cells. **C** and **D.** Morphologic changes were shown in the photographs (C and D top images). Immunoblot analysis of Hela cells express E-cadherin, N-cadherin, and STAT3 levels. Scale bar, 100 μM. All values shown are the mean ± SD of triplicate measurements. Experiments were repeated three times with similar results. (**P < 0.01)

Next, we examined the effect of miR-125a on the invasive abilities of CC cells. We observed that miR-125a overexpression significantly inhibited cell invasion in Hela, SiHa, and CaSki cells using a Matrigel invasion assays (Figure 5A and Supplementary Figure 5A). On the other hand, miR-125a inhibition had the opposite effects in these cell lines (Figure 5B and Supplementary Figure 5B). Re-expression of STAT3 reversed the effects of miR-125a on Hela cell invasion (Figure 5A). MMPs, which degrade extracellular matrix (ECM) proteins and breakdown the tissue barriers to invasion and metastasis, have been thought to be one of the mechanisms for tumor invasion and metastasis. Interestingly, miR-125a overexpression significantly suppressed expressions of MMP-2 and MMP-9 in Hela and CaSki cells (Figure 5A and Supplementary Figure 5A), and re-expression of STAT3 reversed the effects of miR-125a (Figure 5A). On the contrary, miR-125a inhibition increased MMP-2 and MMP-9 expressions (Figure 5B and Supplementary Figure 5B). These data indicate that miR-125a inhibits MMP-2 and MMP-9 expression through suppression of STAT3, thus inhibiting CC cell invasion.

Furthermore, we found in Hela and CaSki cells miR-125a overexpression inhibited morphologic changes from a polarized epithelial phenotype, that performed an elongated fibroblastoid phenotype (Figure 5C and Supplementary Figure 5C), indicating that miR-125a suppresses EMT. Moreover, miR-125a increased expression of the epithelial marker E-cadherin and decreased the mesenchymal marker N-cadherin expression, accompanied by the inhibition of STAT3 protein level (Figure 5C and Supplementary Figure 5C). MiR-125a inhibition had the opposite effects (Figure 5D and Supplementary Figure 5D). The observed miR-125a-mediated phenotype was rescued by STAT3 overexpression (Figure 5C). These data suggest that miR-125a may control CC metastasis through regulation of EMT.

### Knockdown of STAT3 impairs miR-125a functions in CC cells

To further determine if miR-125a exerting its functions was due to the suppression of STAT3, we investigated the difference of above mentioned effects of miR-125a in STAT3 knockdown Hela cells. As expected, STAT3 knockdown almost abolished the ability of miR-125a to inhibit both cell growth and cell cycle in Hela cells (Supplementary Figure 6A–6C). Moreover, the knockdown effects of STAT3 could be rescued by shRNA-resistant of STAT3 expression (Supplementary Figure 6A–6C). These results strongly demonstrate that miR-125a exerts anti-proliferative function through specifically targeting STAT3 *in vitro*. Similar phenomenon was observed for invasion and metastasis ability of miR-125a in STAT3 knockdown Hela cells compared with control cells, indicating that miR-125a inhibits CC cell invasion and metastasis via inhibition of STAT3 (Supplementary Figure 6D and 6E).

### HPV suppresses p53-mediated activation of miR-125a

**Figure 6:**
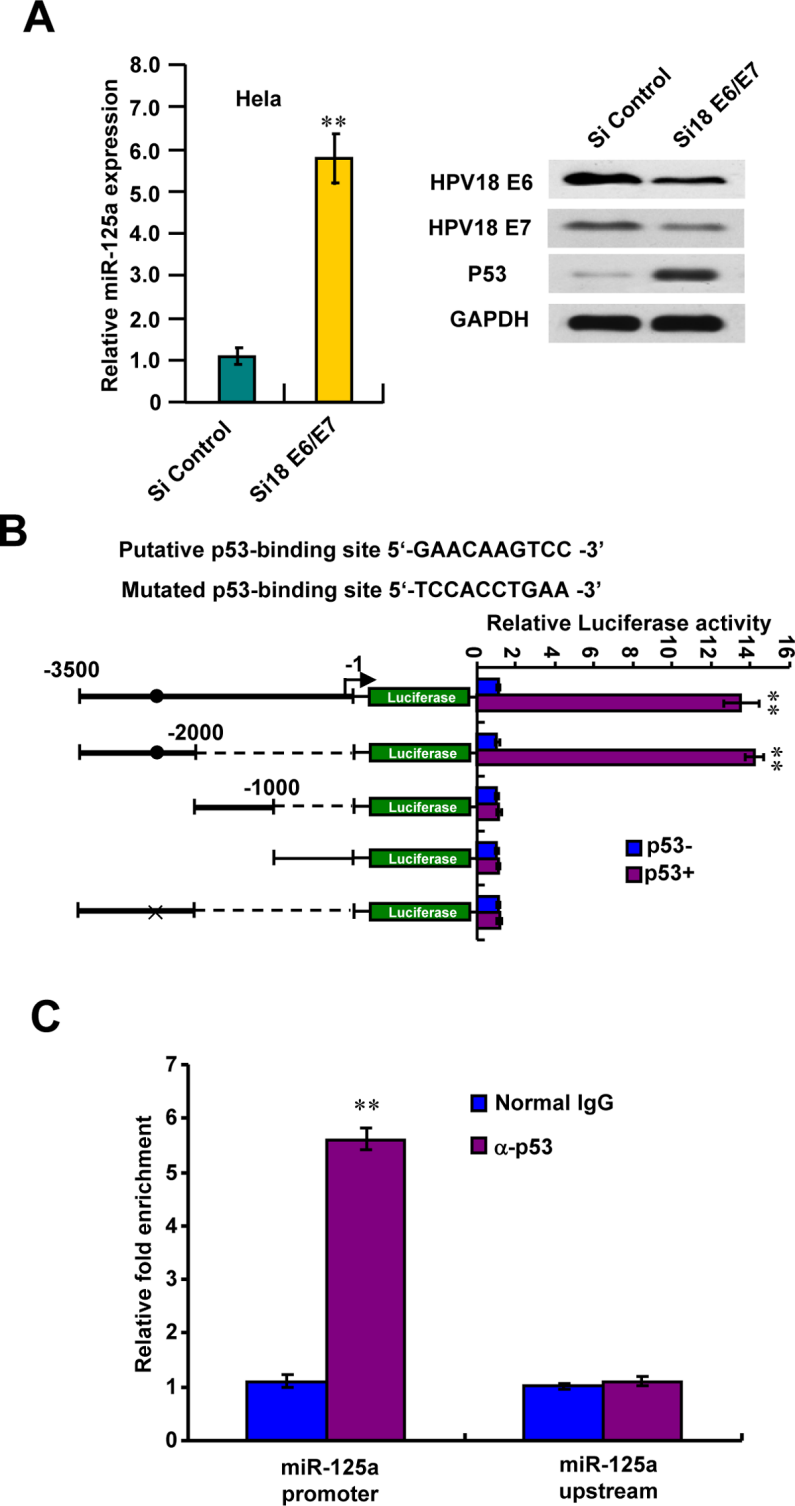
HPV18 E6/E7 suppresses p53-mediated activation of miR-125a **A.** HPV18 E6/E7 knockdown stimulated miR-125a expression. Hela cells were transfected with si18 E6/E7 and analyzed for miR-125a expression by real-time RT-PCR and for HPV18 E6, HPV18 E7 and p53 expression by immunoblot. **B.** Luciferase activity of different promoter constructs in Hela cells transfected with p53 or empty vector. The arrow indicates the position of the transcriptional start site. Filled circles show the position of the p53-binding site, and the “X” shows the mutated p53-binding site. **C.** ChIP analysis of p53 occupancy on the miR-125a promoter in Hela cells. All values shown are mean ± SD of triplicate measurements and have been repeated 3 times with similar results (**P < 0.01).

HPV E6/E7 protein has been shown suppressed many miRNAs expression. To test if miR-125a downregulation in CC cells was due to HPV infection, we transfected Hela cells with HPV18 E6/E7-siRNA (si18 E6/E7) to silence the endogenous HPV18 E6/E7 expression. As expected, knockdown of HPV18 E6/E7 protein could increase miR-125a expression and reconstitution of p53 expression (Figure 6A). At the same time, we found that p53 maybe the transcriptional factor of miR-125a. To prove this, we characterized a putative p53-binding site in the promoter region of miR-125a. We found that p53 strongly stimulated the activity of the luciferase reporter containing the putative p53-binding site but not the reporter with the mutated binding site or without the putative p53-binding site (Figure 6B). We also indicated that p53 was recruited to the miR-125a promoter region including −2533kb to −2542kb, but not to a region approximately −1kb upstream of binding site (Figure 6C). The similar results could also be obtained in SiHa cells expressing HPV16 E6/E7, through silencing of endogenous HPV16 E6/E7 expression by HPV16 E6/E7-siRNA (si16 E6/E7) (Supplementary Figure 7A–7C). Taken together, these data suggest that downregulation of miR-125a in CC probably maybe caused by HPV infection and p53 pathway inactivation.

### MiR-125a inhibits tumor growth and metastasis of CC in nude mice

**Figure 7:**
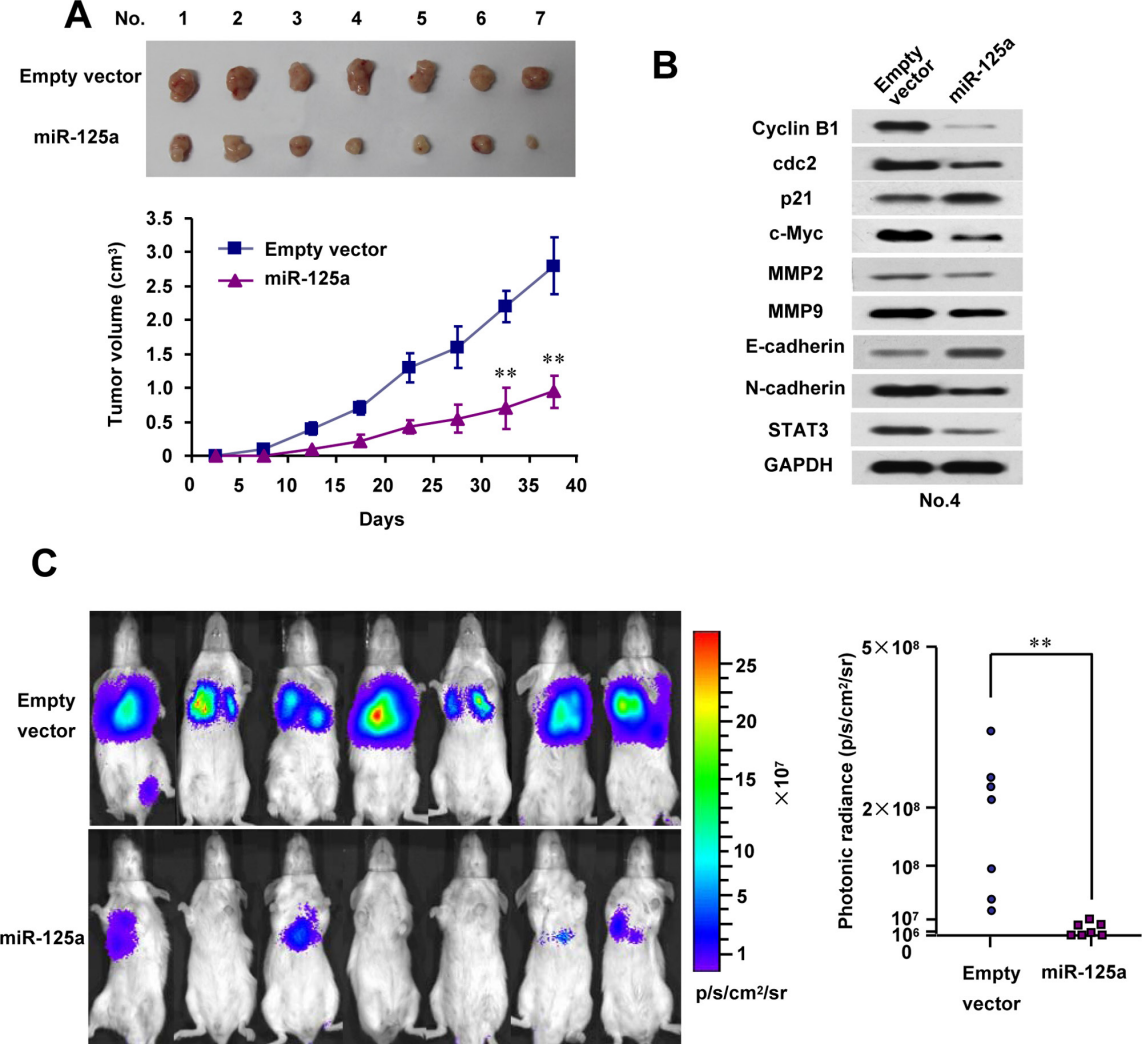
MiR-125a reduces tumor growth and metastasis of CC cell lines *in vivo* **A.** Stable Hela cells overexpressing miR-125a were injected into nude mice. At the indicate times, tumors were measured with Vernier calipers (mean ± SD; *n* = 7). **B.** Immunoblot analysis of representative excised tumors in A. **C.** Bioluminescence imaging of CC cell metastasis in NOD-SCID mice (*n* = 7) at 50 days after intravenous injection of PCDH-control- or PCDH-miR-125a-infected CaSki cells via the lateral tail vein. The luminescence signal is represented by an overlaid false-color image with the signal intensity indicated by the scale. The two-tailed Student's *t*-test was used to compare the photonic radiances in the two groups. (*P < 0.05, **P < 0.01)

To investigate the *in vivo* phenotype of cells overexpressing miR-125a, we first examined the effect of miR-125a on Hela cell growth in nude mice. Consequently, tumor growth was markedly inhibited by miR-125a overexpression (Figure 7A). As expected, the tumors in mice formed by miR-125a-overexpressing Hela cells had reduced levels of STAT3 and its downstream effectors, cyclin B1, cdc2, c-myc, MMP-2, MMP-9, and EMT markers (Figure 7B and Supplementary Figure 8).

Next, we used a metastatic CC cell line, CaSki, to measure the effect of miR-125a overexpression on metastasis. Importantly, mice injected with miR-125a-overexpressing CaSki cells showed a significant decrease in lung metastatic burden compared with the empty vector group (Figure 7C). The intensity of photonic radiance in the lungs of the miR-125a group was much lower than that in the control group (Figure 7C). These findings strongly support the role of miR-125a as a suppressor of tumor dissemination.

### Expression of STAT3 and the correlation between miR-125a and STAT3 in human CC samples

**Figure 8:**
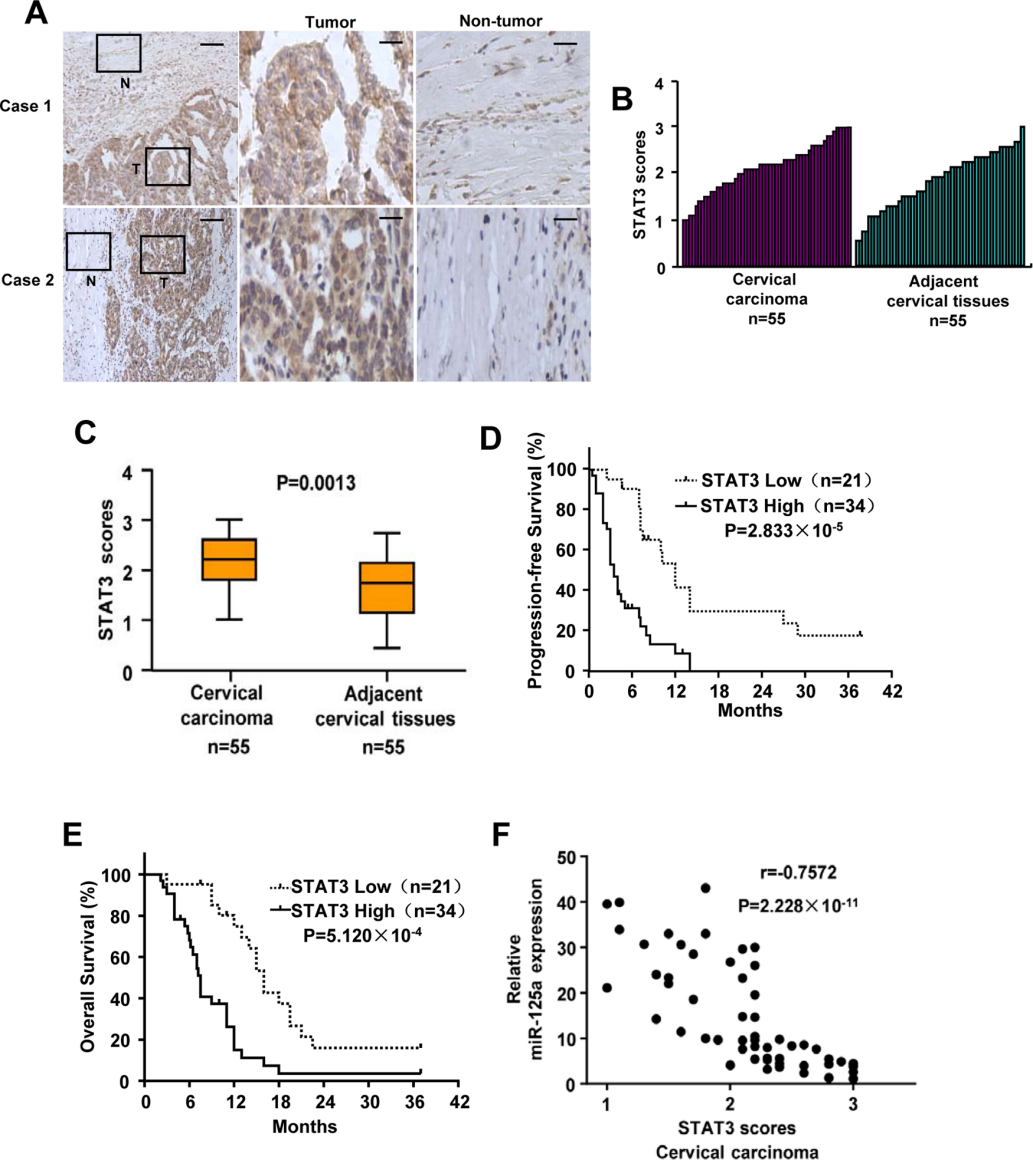
Expression of STAT3 and its correlation with miR-125a in CC patients **A.** Representative immunohistochemical staining of STAT3 in tumorous (T) cervical tissues and adjacent normal (N) cervical tissues. The boxed areas in the left images are magnified in the middle and right images. Scale bars: 250 μm (left) and 50 μm (middle and right). **B** and **C.** STAT3 expression scores were (B) plotted and (C) compared by the Mann–Whitney *U*-test. **D** and **E.** Kaplan–Meier survival curves and the log-rank test were used to assess (D) PFS and (E) OS compared between low and high scores of STAT3 in CC patients. **F.** The relationship between miR-125a and STAT3 expression was assessed by Spearman rank correlation analysis of CC samples. Symbols represent individual samples.

First, we performed Western-blot and qPCR analysis to examine the expression of STAT3 in CC cell lines and normal cervical epithelial cells. The result showed that expression of STAT3 was lower in normal cervical epithelial cells compared with that of CC cells. In addition, CC cells derived from primary cervical cancers expressed lower levels of STAT3 than the ones derived from metastatic sites (Supplementary Figure 9). Second, we assessed STAT3 expression by immunohistochemical staining of tissues including 55 pairs of human cervical carcinomas and their matched adjacent non-tumor cervical tissues. Based on immunohistochemical staining scores, STAT3 expression was significantly upregulated in CC patients (*P* = 0.0013) (Figure 8A–8C). Moreover, Kaplan–Meier survival analysis of STAT3 expression revealed that patients with high STAT3 scores had poorer progression-free survival (PFS) (*P* = 2.833 × 10^−5^) and overall survival (OS) (*P* = 5.120 × 10^−4^) than those with low STAT3 scores, indicating that STAT3 predicts poorer clinical outcome (Figure 8D and 8E). We confirmed the specificity of the anti-STAT3 antibody by preincubation of the antibody with its antigen before immunohistochemical staining (Supplementary Figure 10A) and immunoblotting of lysates from Hela cells transfected with STAT3 shRNA (Supplementary Figure 10B). In agreement with miR-125a inhibition of STAT3 in cultured cells, expression of miR-125a was negatively correlated with STAT3 expression in CC samples (*P* = 2.228 × 10^−11^, *r* = −0.7572) (Figure 8F). Taken together, these data strongly suggest important pathological roles of miR-125a and STAT3 in CC.

## DISCUSSION

Accumulating evidence indicates that suppressed miR-125a expression is a frequent molecular event in human malignances [19–23]. However, the effects of miR-125a dysregulation in CC remain unknown. In the present study, we showed that low expression of miR-125a was more frequent in cervical cancer tissues than in adjacent normal tissues. Our result showed that miR-125a downregulation was significantly associated with large tumor size (≥4 cm), late FIGO Stage (III/IV Stage), and metastasis situation (lymphatic metastasis and distant metastasis). It has been widely acknowledged that smaller tumor size and early stage of cervical cancer have a more favorable prognosis while metastasis situation has poorer prognosis. Our successive survival analysis result revealed that low expression of miR-125a had shorter PFS and OS. Therefore, the correlation between miR-125a expression and pathological variables is consistent with the prognostic significance of miR-125a in CC patients.

MiR-125a was a negative regulator of CC cell growth and invasion *in vitro* and *in vivo*. In addition, we identified protumorigenic effects of STAT3 as a target of miR-125a. We further demonstrated that miR-125a suppressed cell growth by promoting activation of cell cycle arrest, which was accompanied by changes in the expression of G2/M checkpoint proteins and c-myc via downregulation of STAT3 expression. In addition, miR-125a inhibited cell invasion by decreasing MMP-2, MMP-9, and N-cadherin, increasing E-cadherin expression through STAT3. Finally, miR-125a was negatively correlated with STAT3 expression that was upregulated in patients with CC. Therefore, miR-125a might play an important role in the development and progression of CC through regulation of STAT3 expression.

STAT3 upregulation in cancers contribute to tumorigenesis and metastatic behavior. STAT3 upregulation has pro-tumorigenic functions that enhance tumor cell growth, invasion and metastasis in a variety of cancers [24–26]. Meanwhile, high STAT3 expression and STAT3 signal activated have be reported in many cancers, and its expression generally received a worse prognosis in terms of both PFS and OS [24–26]. In consistent with the previous findings in CC tissues [26, 27], the results obtained from our study demonstrated that STAT3 expression was significantly increased and the high expression level was correlated with a more aggressive behavior of the disease. Therefore, inhibition of STAT3 may serve as a potential method for therapeutic against CC.

Exploring the molecular mechanisms of cancer cell growth is critically important. It is widely accepted that cell cycle dysregulation is a major factor in cancer cell growth [18, 28]. Interestingly, upon activation of the JAK2-STAT3 pathway, STAT3 promotes cyclin B1 [29, 30] and cdc2 [29] expression, and inhibits p21 [29] and p53 [29] expression. Furthermore, upregulation of STAT3 in cancers can contribute to cell growth. For example, STAT3 overexpression has protumorigenic effects that enhance cell growth in a variety of tumors [31, 32]. Similar to these previous findings, our results demonstrated that STAT3 expression was significantly increased in CC tissues. Our functional analyses showed that miR-125a directly targeted STAT3 and inhibited its expression. This regulation led to inhibition of cell proliferation by miR-125a overexpression. Moreover, a high expression level of miR-125a was correlated with less aggressive behavior of the tumor. In addition to cell cycle arrest, miR-125a downregulated expression of the oncogene c-myc through inhibition of STAT3, which might be another molecular mechanism through which miR-125a suppresses CC cell growth. Therefore, miR-125a may serve as a potential target for therapeutic intervention against CC.

Invasion and metastasis are major reasons for the poor prognoses of CC patients. Previous studies have indicated that many molecular mechanisms contribute to the metastasis of CC, including epithelial–mesenchymal transition [33], MMP up-regulation [34] and/or down-regulation of TIMPs (tissue inhibitor of metalloproteinases) [34], and metastasis due to vascular endothelial growth factor up-regulation [35]. During metastasis, EMT can reduce the adhesion between cancer cells and enhance its ability to metastasis. MMPs play critical roles in degradation of the extracellular matrix for cancer cells invading from primary sites to other tissues and organs. E-cadherin, N-cadherin, MMP-2 and MMP-9 have been found to be related to CC metastasis [12, 34, 36]. In this study, we showed that miR-125a overexpression modulated the invasion and metastasis of CC cells, along with decreased expression of N-cadherin, MMP-2, MMP-9 and increased expression of E-cadherin. More interestingly, miR-125a directly targeted STAT3 that acts as a transcription factor for these genes [34, 37], suggesting that miR-125a regulated CC metastasis by inhibiting STAT3-mediated EMT and MMPs expression.

In our study, we observed down-regulation of miR-125a in CC tissues. There were several possible reasons for the phenomenon. First, miR-125a is located on chromosome 19q13.41 in which alterations of the genomic region may be responsible for the down-regulation of miR-125a. Second, miR-125a down-regulation might be due to epigenetic silencing via DNA methylation and/or chromatin histone deacetylation. Lastly, HPV infection may regulate the expression of many miRNAs including miR-125a. HPV might regulate miR-125a expression by increasing DNA methylation of its promoter or interacting with transcription factors that regulate miR-125a expression. These mechanisms are common regulatory pathways through which HPV regulates other genes [9]. In our study, we found that both HPV18 E6/E7 and HPV16 E6/E7 could inhibit miR-125a expression. We also confirmed previous studies result that HPV18 E6/E7 and HPV16 E6/E7 could increase p53 expression. P53, as a transcription factor, bound to the promoter of miR-125a, and stimulated its transcription. So we speculated the reason of low expression of miR-125a in CC was due to the suppression of p53 caused by HPV infection. However, this might not be the only molecular mechanism. Further studies are still needed to evaluate other mechanisms of miR-125a down-regulation in CC.

In conclusion, reduced miR-125a expression frequently exists in CC. MiR-125a inhibits the tumorigenesis and metastasis of cervical tumors by targeting STAT3. Activation of miR-125a or inhibition of STAT3 may be useful strategies for CC treatment.

## MATERIALS AND METHODS

### Patients and tumor tissues

A total of 55 human cervical cancer samples and matched normal adjacent cervical tissues were obtained from the Chinese PLA 309th Hospital with the informed consent of patients and approval for experimentation from the Chinese PLA 309th Hospital and PLA General Hospital. Diagnoses were based on pathological evidence. None of the patients had undergone immunotherapy, chemotherapy, hormone therapy, or radiotherapy before specimen collection. The clinical stage and histological grades were based on FIGO. Tissue samples were divided into two portions with one snap-frozen immediately in liquid nitrogen and stored in −80°C until RNA extraction, while the other portion was used for histopathological diagnosis. Clinical and pathological data of the patients are supplied in Table 1

### Cell culture and transfection

Cervical cancer cells, including Hela (Adenocarcinoma), SiHa (Squamous cell carcinoma), ME-180 (Epidermoid carcinoma), CaSki (Epidermoid carcinoma), C-33A (Epidermoid carcinoma), SW756 (Squamous cell carcinoma), and MS-751 (Epidermoid carcinoma) cell lines were obtained from the American Type Culture Collection (Manassas, VA, USA). Human normal cervical epithelial cells were bought from CHI Scientific, Inc (Maynard, MA, USA). Stable cell lines overexpressing miR-125a and STAT3 shRNAs were established by lentiviral transduction using a pCDH plasmid (System Biosciences, Mountain View, CA, USA) carrying miR-125a or STAT3 shRNAs. For transfection [38], cells were seeded in 24- or 6-well plates and then transfected with the indicated plasmids using Lipofectamine 2000 (Invitrogen) according to the manufacturer's protocol.

### Plasmid construction and reagents

The expression vector for the miR-125a precursor sequence was generated by cloning the PCR product into a pcDNA3.1 vector (Invitrogen) or pCDH plasmid (System Biosciences) using the following primers: 5′-CGGGATCCTCTTTCTGTCTCTGGCTCTCAGAA-3′ (forward) and 5′-CGGAATTCAGTGGTCTGGGGTCA GAGGTCA-3′ (reverse). The antisense miR-125a oligonucleotide (anti-hsa-miR-125a) and antisense miRNA control were purchased from Qiagen (Valencia, CA, USA). Wild-type and mutated STAT3 3′-UTRs, including the predicted target sites of miR-125a, were cloned into a pmir-GLO dual-luciferase reporter vector (Promega, Madison, WI, USA). To obtain the wild-type STAT3 3′-UTR, the following primers were used to amplify the gene sequence: 5′-CGGAATTCAGGAATCCTGGTCTCAGGACCTC-3′ (forward) and 5′-GCTCTAGATCATACGAGGGCAG ACTCAAGT-3′ (reverse). To introduce mutations into the target sites within the STAT3 3′UTR for seed sequences of the predicted miR-125a, recombinant PCR was performed using the abovementioned primers and the following primers: 5′-TGGGGCCCCAGCGACGTGTCTGGTTGAGAGA CTTTCA-3′ (forward) and 5′-GAAAGTCTCT CAACCAGACACGTCGCTGGGGCCCCA-3′ (reverse). The expression vector for STAT3 shRNA and expression constructs for shRNA-resistant STAT3 were purchased from GenePharma (Shanghai, China). Expression vectors for HPV18 E6/E7 and HPV16 E6/E7 have been previous described [39].

Anti-cyclin B1, anti-cdc2, anti-p53, anti-HPV18 E6, anti-HPV18 E7, anti-HPV16 E6, anti-HPV16 E7 and anti-GAPDH antibodies were purchased from Santa Cruz Biotechnology (Dallas, TX, USA). Anti-MMP-2 and anti-MMP-9 antibodies were obtained from Cell Signaling Technology (Boston, MA, USA). Anti-p21 and anti-c-myc antibodies were purchased from Proteintech (Chicago, IL, USA). The anti-STAT3 antibody was purchased from Abcam (Cambridge, MA, USA).

### Luciferase reporter assay

CC cells were seeded into 24-well plates. Wild-type or mutant STAT3 3′UTR reporter constructs were co-transfected with miR-125a into the cells with Lipofectamine 2000. At 48 h after transfection, the cells were harvested and analyzed for luciferase activity using a luciferase assay kit (Promega) according to the manufacturer's protocol.

### ChIP

ChIP assay was performed with Hela and SiHa cells using the Magna ChIP Assay Kit (EMD Millipore, Temecula, CA, USA) according to the manufacturer's instructions. Protein-DNA complexes were precipitated with normal IgG and anti-p53 (Santa Cruz) at 4°C overnight with rotation. PCR was performed with the following primers: miR-125a promoter sense, 5′-TATGGGCCCAGGGAGTTCGCGTTT-3′; miR-125a promoter antisense, 5′-GAGATTCCCCGGAC CTAAGCATCT-3′; miR-125a upstream sense, 5′-AGGTGTGCCCAAAGGGCCAAATTA-3′; miR-125a upstream antisense, 5′-CAGACTCATGAGTCCAGA TCCAAA-3′.

### Cell proliferation and colony formation assays

Cell proliferation was determined by anchorage-dependent and -independent cell growth. For anchorage-dependent cell growth, transfected cells were seeded in 96-well plates and analyzed at 0, 24, 48, 72 and 96 h using a CCK-8 Kit (Dojindo, Kumamoto, Japan) according to the manufacturer's protocol. For anchorage-independent cell growth, a bottom layer of 0.7% low melting temperature agar and a top layer of 0.35% agar mixed with transfected cells were plated in 6-well plates. Colonies with diameters greater than 100 μm were counted after 3 weeks of growth. For colony formation assays, transfected cells were seeded in 6-well plates at 2, 000 cells per well. Two weeks later, the colonies were fixed with 4% paraformaldehyde and stained with a crystal violet solution for 30 min. The number of colonies containing at least 50 cells was counted.

### Cell invasion assays

Cell invasion assays used transwell chambers (Corning; Tewksbury, MA, USA) coated with Matrigel (BD Biosciences; San Jose, CA, USA) on the upper surface. Briefly, cells were seeded in the chambers with medium containing 0.1% FBS, while medium containing 20% FBS was placed in the lower well. Twenty-four hours later, cells that invaded through the Matrigel were fixed with 4% paraformaldehyde and stained with crystal violet. The number of invaded cells was counted in five randomly selected microscopic fields and photographed.

### Cell cycle analysis

Transfected cells were fixed in 70% ethanol for 24 h, and then washed twice with PBS. The cells were incubated with RNase A (1 mg/mL) in PBS containing 0.1% bovine serum albumin (Sigma-Aldrich, St. Louis, MO, USA) for 30 min at 37°C. Propidium iodide (Sigma-Aldrich) was then added to the cell suspension. After 30 min of incubation at room temperature while protected from light, the samples were analyzed by a FACSCalibur Flow Cytometer (Becton Dickinson, Franklin Lakes, NJ, USA).

### *In vivo* tumor growth and metastasis

Female 6-week-old BALB/c nu/nu and nonobese diabetic-severe combined immunodeficient (NOD-SCID) mice were purchased from Vital River Inc. (Beijing, China). For the tumor growth model, Hela cells labeled with firefly luciferase and stably transfected with the pCDH control vector or pCDH-miR-125a were injected subcutaneously into the backs of BALB/c nu/nu mice (*n* = 7). Tumor sizes were measured at the indicated times using calipers. Tumor volumes were estimated according to the following formula: volume = (longest diameter × shortest diameter^2^)/2. For the metastasis model, 1 × 10^6^ CaSki cells labeled with firefly luciferase and stably transfected with pCDH control or pCDH-miR-125a were injected intravenously via the lateral tail vein of NOD-SCID mice (*n* = 7). All mice were maintained for about 50 days until analysis by the IVIS200 imaging system (Xenogen Corporation, Alameda, CA, USA).

### MiRNA extraction and quantitative RT-PCR

Total RNA was extracted from cultured cells or tissues samples with an miRNeasy Mini kit (Qiagen). The cDNA of target miRNA was reverse transcribed from the total RNA using a specific miRNA primer and miScript Reverse Transcription Kit (Qiagen). MiRNA expression was measured with a miScript SYBR Green PCR Kit (Qiagen) using the ABI7500 Real-Time PCR System (Applied Biosystems, Foster City, CA, USA). Primers for endogenous control U6 and miR-125a were purchased from Qiagen. The relative fold expression of the target was calculated by the comparative Ct method and normalized to the control.

### Immunohistochemical analysis

Briefly, antigens were retrieved by microwave treatment, and then the samples were incubated with a rabbit anti-STAT3 antibody (Abcam) at a dilution of 1/200. Bound primary antibodies were detected by addition of a biotinylated goat anti-rabbit secondary antibody and streptavidin-horseradish peroxidase. The stained samples were developed with 3, 3′-diaminobenzidine and counterstained using hematoxylin. For negative controls, PBS was substituted for the primary antibody. All immunohistochemical staining was assessed by two pathologists blinded to the specimen information. The widely accepted H-score system was employed to assess the staining intensity (0–3: 0, no staining; 1, weak staining; 2, moderate staining; 3, strong staining) and the percentage of positively stained cells (0–100%). Each individual intensity level was the summation of the product of the staining intensity and the proportion of stained cells to obtain the final H-score.

### Statistical analysis

All *in vitro* experiments were performed in triplicate and repeated three times. The differences in miR-125a or STAT3 expression between CC and adjacent cervical tissues were assessed by the Mann–Whitney *U*-test. The relationships between miR-125a and clinicopathological parameters were evaluated by the χ^2^ test. The survival rates in relation to miR-125a expression were estimated by the Kaplan-Meier method, and the difference in survival curves was tested by the log-rank test. Statistical differences in cell proliferation and invasion assays among mutant constructs was determined by the two-tailed Student's *t*-test. The relationship between miR-125a and STAT3 expression was explored by the Spearman rank correlation. The SPSS 17.0 statistical software package was used to perform all statistical analyses. Data are presented as the means ± standard deviation (SD). *P* < 0.05 was considered statistically significant.

## SUPPLEMENTARY FIGURES


