# Supplementary material for: MiR-125a suppresses tumor growth, invasion and metastasis in cervical cancer by targeting STAT3
Source: Oncotarget. 2015 Jul 13;6(28):25266–80. doi: 10.18632/oncotarget.4457 (PMC4694830; doi:10.18632/oncotarget.4457)
Supplement: Supplementary file 1 [file oncotarget-06-25266-s001.pdf]

SUPPLEMENTARY FIGURES

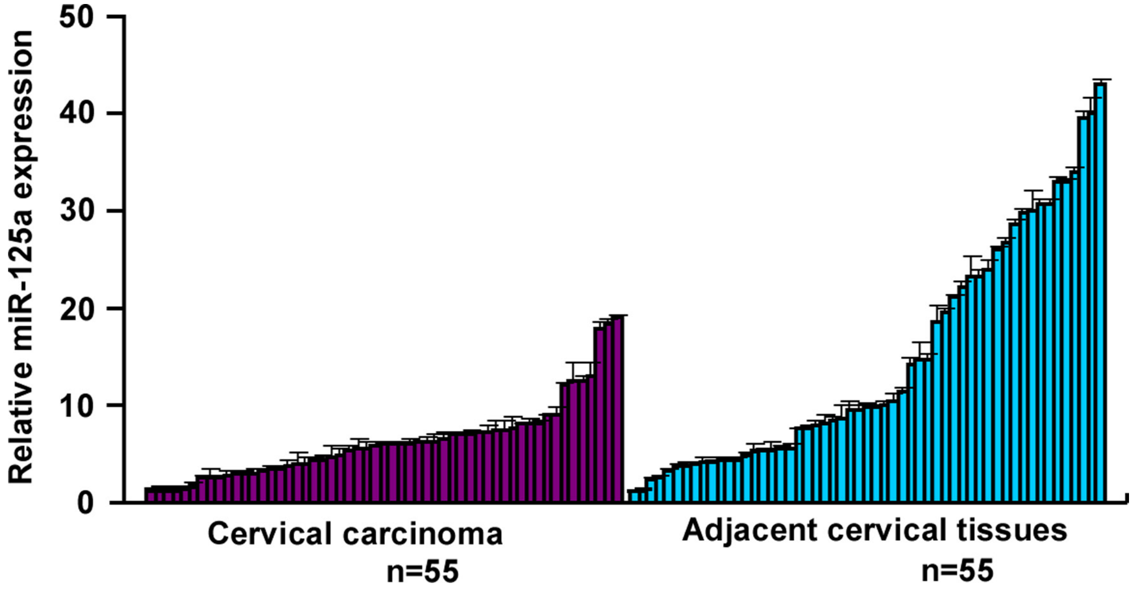

Supplementary Figure S1: Expression of miR-125a in CC. Expression of miR-125a in CC tissues and matched adjacent normal cervical tissues (*n* = 55) was assessed by real-time RT-PCR.

A

Target gene	ResfSeq Id
SP1	NM_138473
RET	NM_020630
TP53INP1	NM_001135733
EIF4EBP1	NM_004095
STAT3	NM_003150
MSI1	NM_002442

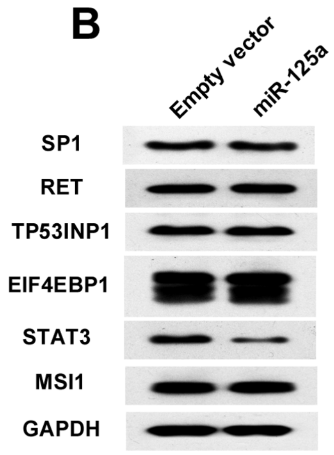

Supplementary Figure S2: Potential target gene of miR-125a screened. A. Candidate target genes of miR-125a were found using publicly available databases (TargetScan and miRanda). **B.** Immunoblot analysis protein levels of the candidate target genes in Hela cells transfected with miR-125a.

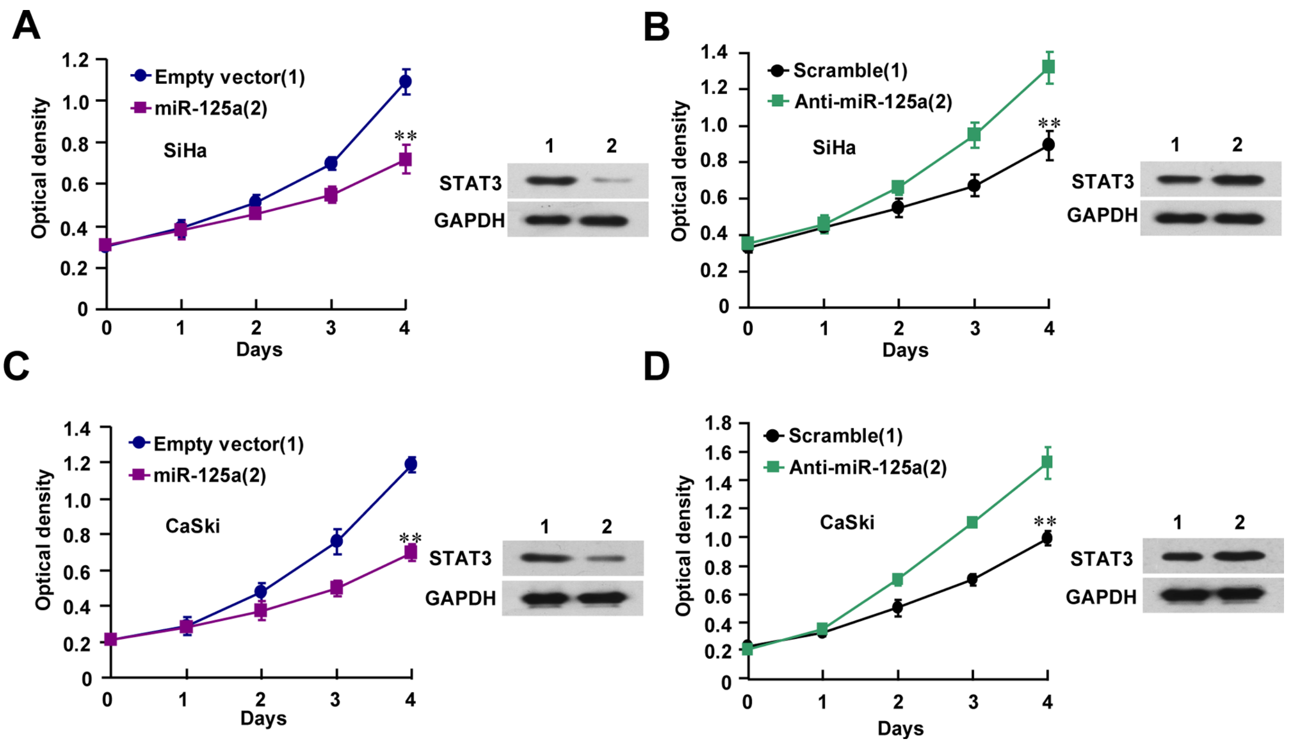

Supplementary Figure S3: Mir-125a reduces CC cell growth. A–D. SiHa cells (A and B) and CaSki cells (C and D) expressing miR-125a (A and C) or anti-miR-125a (B and D) were grown in regular medium. At specified times, cell numbers were determined by CCK-8 assay. Representative Western blot shows STAT3 expression (A-D right panels). All values shown are mean ± SD of triplicate measurements and have repeated 3 times with similar results (***P* < 0.01 versus empty vector or scramble vector)

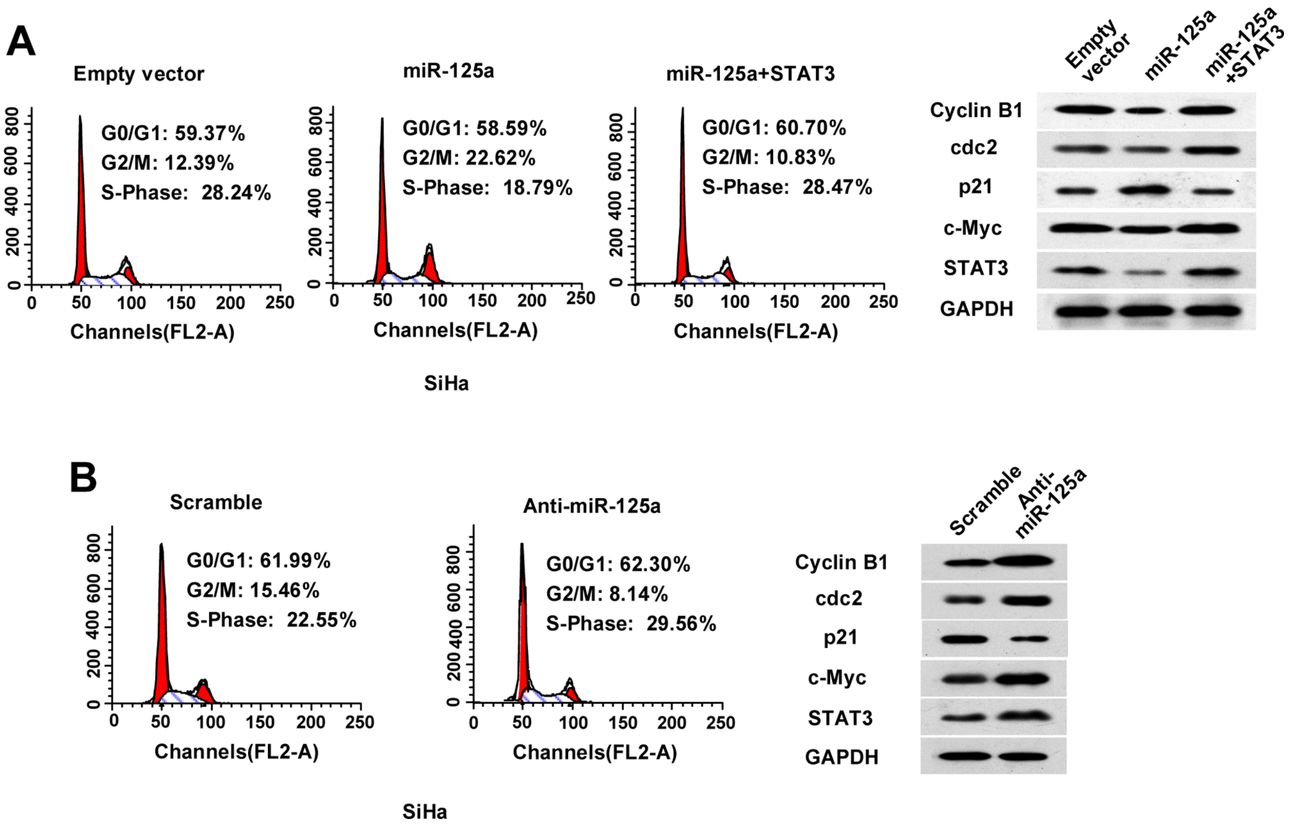

Supplementary Figure S4: Mir-125a decreases the G2/M transitions in CC cells. A and **B.** Flow cytometry analysis of cell cycle in SiHa cells transfected with (A) miR-125a of miR-125a plus STAT3 (B) or anti-miR-125a. The experiments have been repeated three times with similar trends and the images displayed is one of the representative results. Representative Western blots for Cyclin B1, cdc2, p21, c-myc and STAT3 proteins in above cells (right panels).

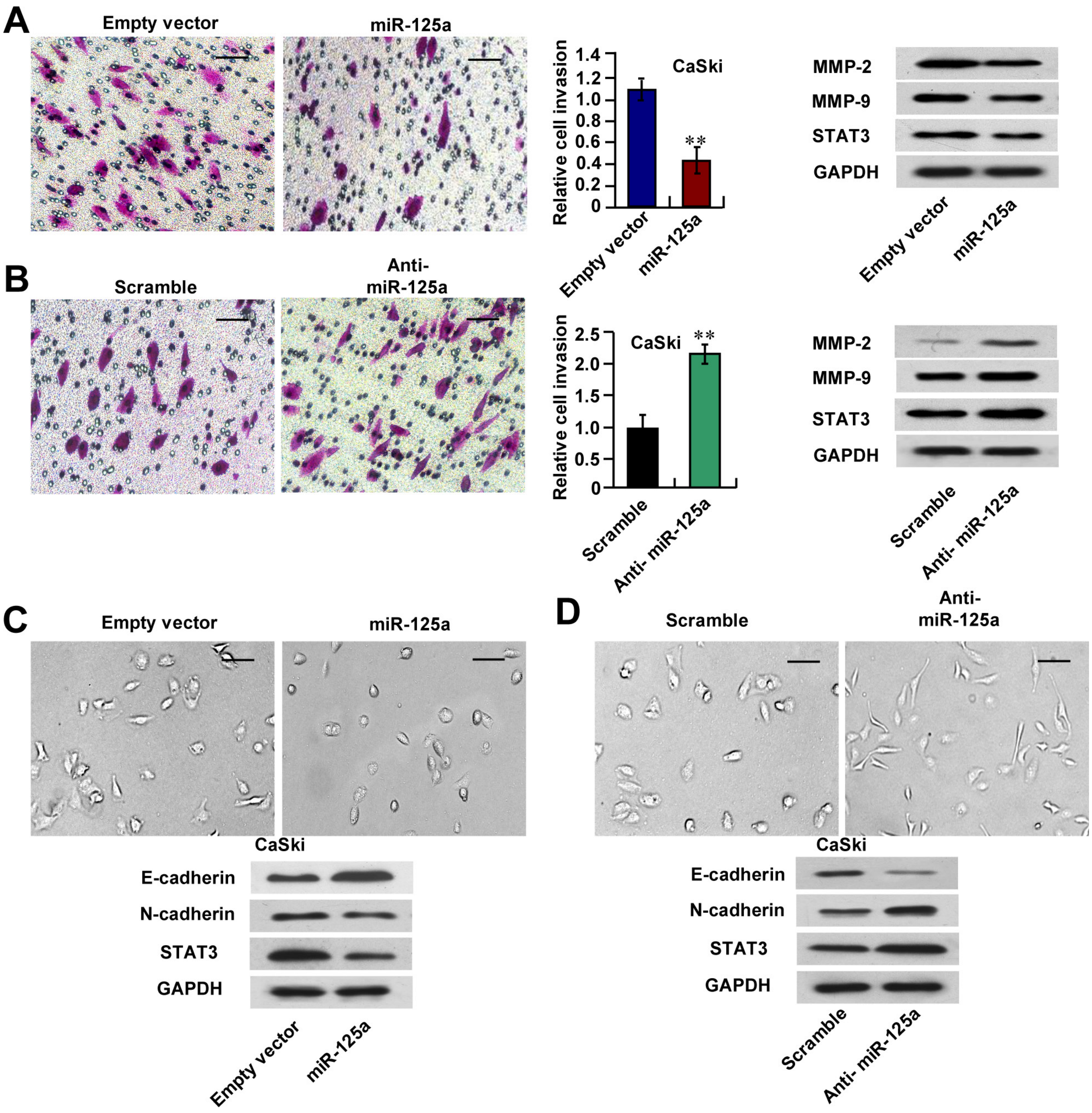

Supplementary Figure S5: Mir-125a inhibits invasion in CaSki cells. A and **B.** Cell invasion was evaluated in CaSki cells expressing (A) miR-125a or (B) anti-miR-125a using a Matrigel invasion chamber. Invasive cells were fixed and stained with crystal violet (left images). Scale bar, 100 µM. Middle histograms show relative cell invasions. Western blots for MMP-2, MMP-9 and STAT3 proteins with indicated CaSki cells (right panels). **C** and **D.** Morphologic changes are shown in the photographs in CaSki cells expressing (C) miR-125a or (D) anti-miR-125a. Western blots for EMT markers proteins were shown (lower panels). All values shown are mean ± SD of triplicate measurements and have been repeated 3 times with similar results. (***P* < 0.01).

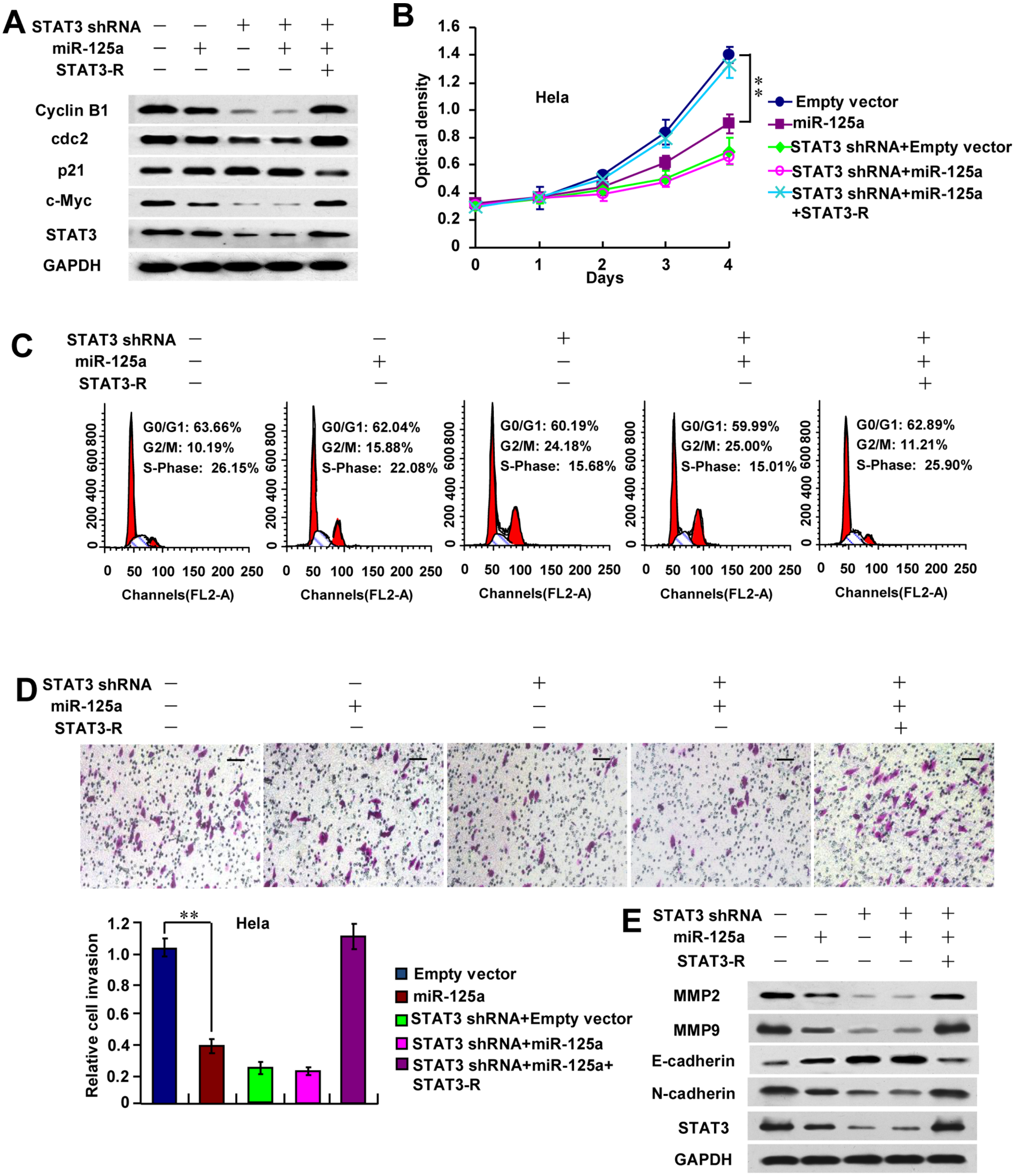

Supplementary Figure S6: Mir-125a represses cell proliferation and invasion through inhibition STAT3 expression. Hela cells expressing miR-125a, STAT3 shRNA, miR-125a plus STAT3 shRNA, or miR-125a together with STAT3 shRNA and shRNA-resistant STAT3(STAT3-R) were cultured in regular medium. **A** and **E.** Immuniblot analysis of Hela cells. **B.** At specified times, cell numbers were determined by CCK-8 assay. **C.** Cell cycle was evaluated using Flow cytometry analysis. **D.** Cell invasion was evaluated using a Matrigel invasion chamber. Scale bar, 100 μM. All values shown are mean ± SD of triplicate measurements and have been repeated 3 times with similar results. (**$P < 0.01$).

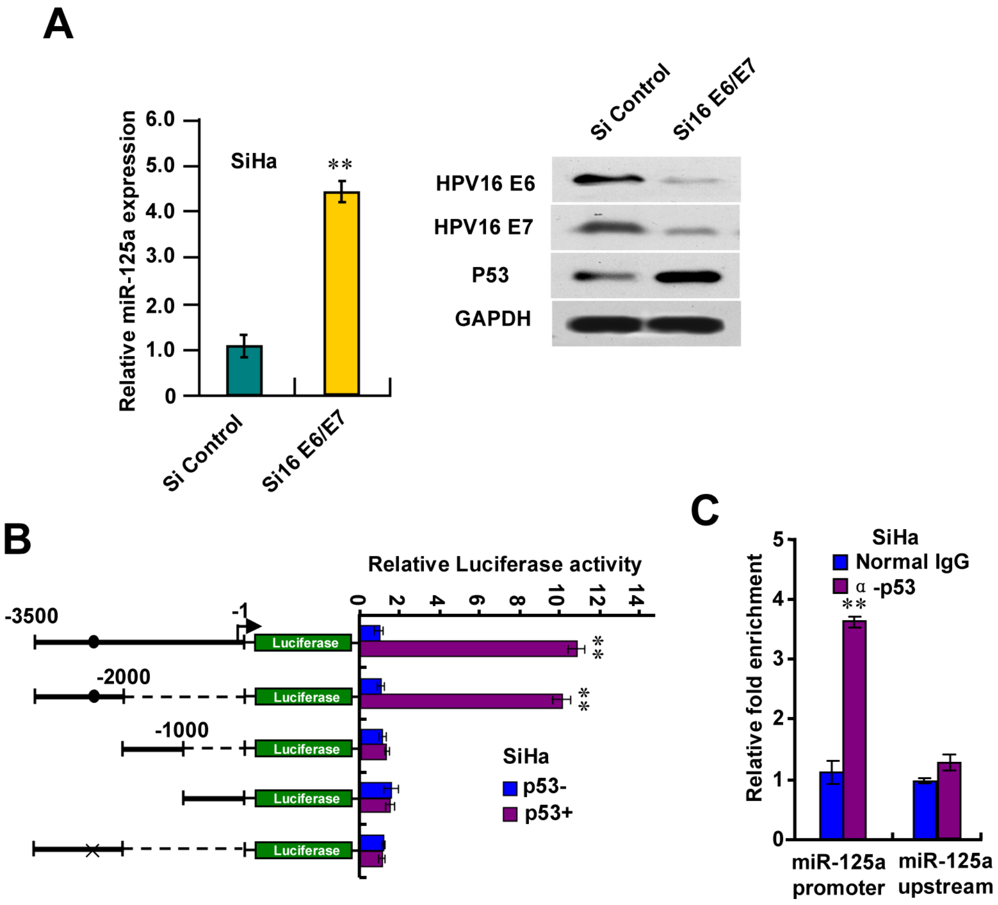

Supplementary Figure S7: HPV16 inhibited miR-125a expression through p53-mediated inactivated. A. HPV16 suppresses miR-125a expression. SiHa cells were knockdown HPV16 E6/E7 expression and analyzed for miR-125a expression by RT-PCR and for p53 expression by immunoblot. **B.** Luciferase activity of different promter constructs in SiHa cells transfected with p53 or empty vertor. The arrow indicate the position of the transcriptional start site. Filled circle show the position of the p53-binding site, and the "**X**"shows the mutated p53-binding site. **C.** ChIP analysis of p53 occupancy on the miR-125a promoter in SiHa. All values shown are mean ± SD of triplicate measurements and have been repeated 3 times with similar results. (***P* < 0.01).

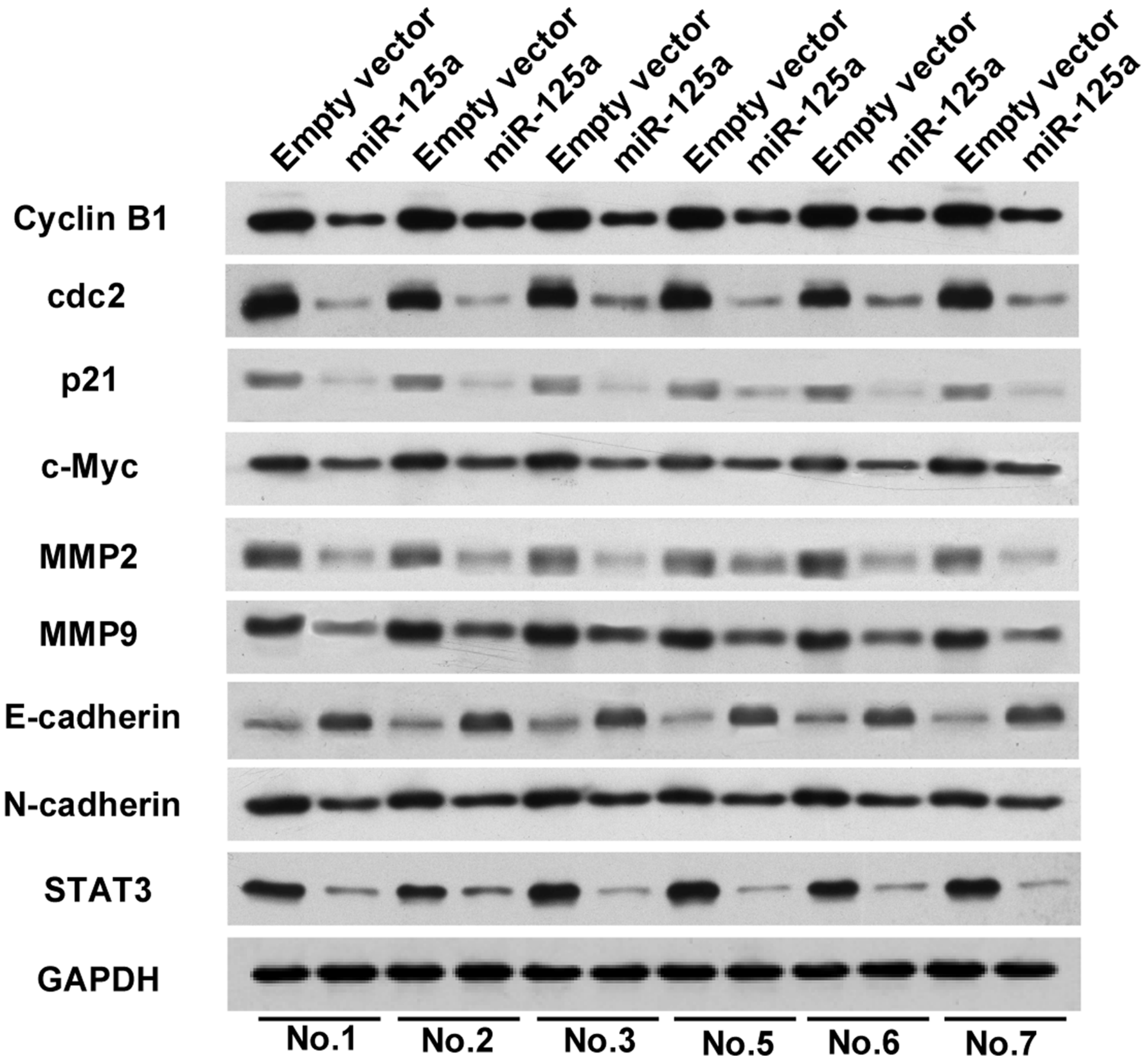

Supplementary Figure S8: miR-125a reduces STAT3 and downstream effectors in mice tumor mass. Immunoblot analysis of tumor masses (No. 1–3 and No. 5–7) formed by miR-125a-overexpressing Hela cells in mice.

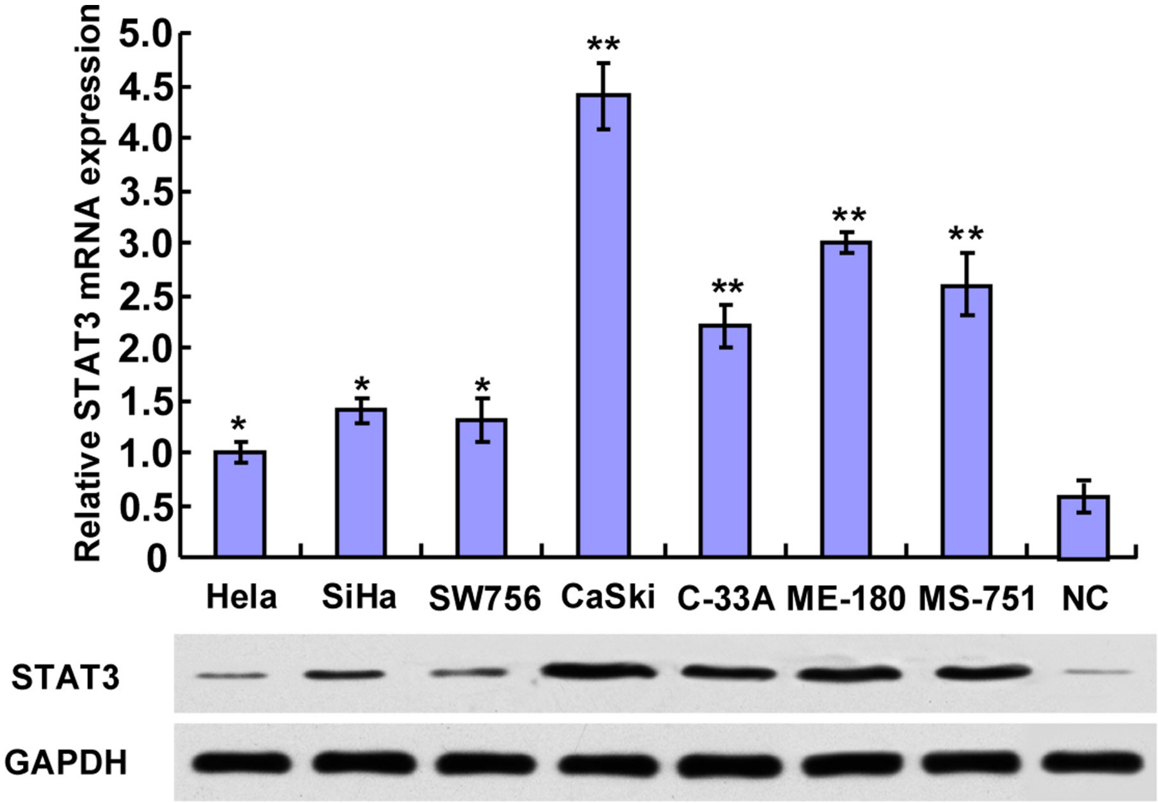

Supplementary Figure S9: Expression of STAT3 in cervical cell lines. Expression of STAT3 in CC cell lines was assessed by real-time RT-PCR and Western blot. All values are the mean ± SD of triplicate measurements, and experiments were repeated 3 times with similar results. $*P < 0.05$, $**P < 0.01$ compared to normal cervical cells.

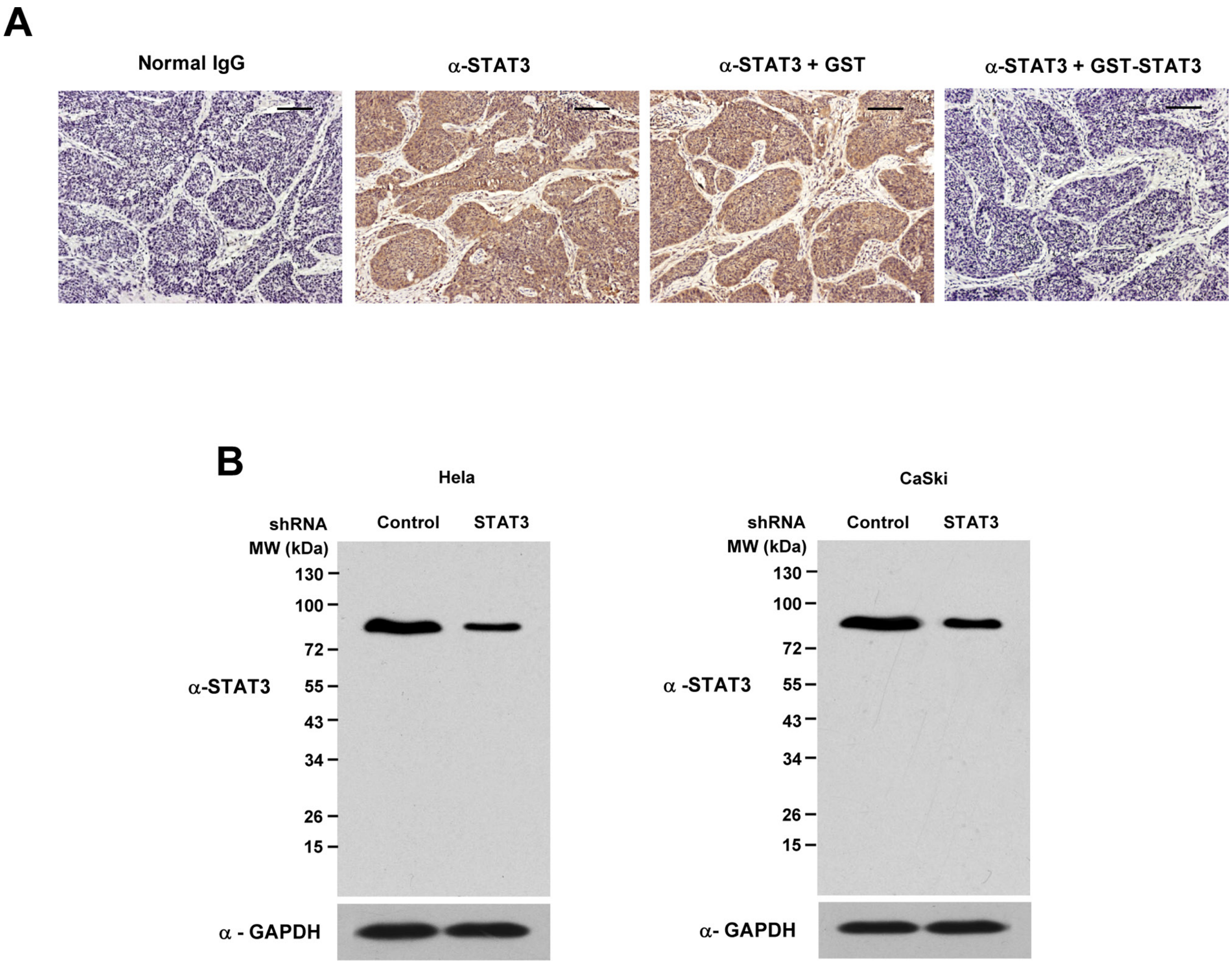

Supplementary Figure S10: Validation of antibody specificity to STAT3. A. Immunohistochemical staining of CC samples incubated with normal IgG or anti-STAT3. To validate antibody specificity, the anti-STAT3 was pre-incubated with recombinant GST-STAT3 protein or GST for 1 h prior to applying to tissue. Original magnification, × 10. Scale bar, 100 µm. **B.** Immunoblot analysis of lysates from Hela (left panel) or CaSki (right panel) cells infected with control shRNA or STAT3 shRNA using antibodies specific for anti-STAT3. MW, molecular weight.
